# Molecular evidence for potential transovarial transmission of Dabieshan tick virus in *Haemaphysalis longicornis* from Shandong Province, China

**DOI:** 10.1371/journal.pone.0296213

**Published:** 2023-12-22

**Authors:** Anan Wang, Yunfeng Tang, Zheng Pang, Yaxuan Gong, Jintao Wu, Jun Qi, Guoyu Niu

**Affiliations:** 1 School of Public Health, WeiFang Medical University, Weifang, China; 2 Tianjin Customs Port Out-Patient Department, Tianjin International Travel Healthcare Center, Tianjin, China; 3 Yantai Zhifu District Center for Disease Control and Prevention, Yantai, China; University of Pécs: Pecsi Tudomanyegyetem, HUNGARY

## Abstract

Dabieshan tick virus (DBTV) is a newly identified arbovirus, first detected in *Haemaphysalis longicornis* collected from Hubei Province in 2015. It has been confirmed that DBTV is widely distributed in Shandong Province, China. However, its entomological and epidemiological features remain to be further explored, particularly the feasibility of transovarial transmission. Our research tries to explain the possibility of transovarial transmission of DBTV from engorged female ticks to their offspring. All engorged female adult ticks were sampled from domestic sheep and allowed to lay eggs and hatch in appropriate laboratory conditions. All engorged ticks, larvae and unhatched eggs were classified into pools for nucleic acid extraction and DBTV RNA detection. According to the results of qRT-PCR, the positive rate of DBTV was 6.25% (8/128) in engorged female ticks, 3.57% (1/28) in eggs and 5% (3/60) in larvae pools, respectively. Phylogenetic analysis indicated that DBTV isolates from larvae were similar to those from maternal ticks with more than 99.5% homology, and DBTV was relatively conservative in evolution. Our findings are the first to provide molecular evidence of potential transovarial transmission of DBTV among *H*. *longicornis*. Nonetheless, the transovarial transmission of DBTV in frequency and proportion occurring in nature deserves further investigation.

## Introduction

Ticks, as the primary arthropod vectors of pathogen transmission, can transmit a wide range of arboviruses. It has been shown that a number of arboviruses have a significant impact on public health and can cause a range of symptoms in humans or animals, from mild to severe, and even death, for instance, in the last two decades, severe fever with thrombocytopenia syndrome virus and Alongshan virus in China [1, 2], Heartland virus and Bourbon virus in the United States [3, 4], Hunter island group virus in Australia [5], Yamaguchi virus, Muko virus and Tarumizu tick virus in Japan [6–8], Shibuyunji virus in Zambia [9], Dugbe virus in Ghana [10]. In recent years, with increased attention and the development of next-generation sequencing technology, several novel arboviruses have been identified. However, the molecular biological characteristics and pathogenicity of most emerging arboviruses have not yet been elucidated. Therefore, it is necessary to conduct comprehensive studies on the identification, investigation, and pathogenicity research of emerging TBVs.

Dabieshan tick virus (DBTV) was initially identified in *H*. *longicornis* from Hubei Province, China, in 2015 [11]. In the literature, DBTV is considered an intermediate species that shares close evolutionary relationships with Yongjia tick virus 1 and Uukuniemi virus (Bunyavirales; Phenuiviridae; Uukuvirus). DBTV has shown a relatively conservative evolution [11, 12]. It had been demonstrated that DBTV was widely distributed in Shandong Province in our previous study [12, 13]. However, there is limited knowledge regarding the ecology of the arthropod vector and the transmission mode of this virus.

Viruses can be transmitted through various routes in ticks. The currently proven routes of virus transmission include horizontal transmission (from host to tick and tick to host), transstadial transmission (from one tick stage to another), transovarial transmission (from female ticks to eggs), mating, and co-feeding, etc [14, 15]. Transovarial transmission plays an extremely important role as a route for some viruses, including the Crimean-Congo hemorrhagic fever virus (CCHFV) [16, 17]. After feeding on the blood of an infected host, a female tick that has mated drops off to lay eggs. This process allows the virus to potentially infect the offspring ticks. Currently, there is limited research on the transmission mode of DBTV. Therefore, this study focuses on the presence of DBTV in engorged female ticks and their offspring collected from the same farm in a DBTV-endemic region of Shandong Province to examine the propensity of this virus to undergo potential transovarial transmission.

## Materials and methods

### Tick collection and egg incubation

**Fig 1:**
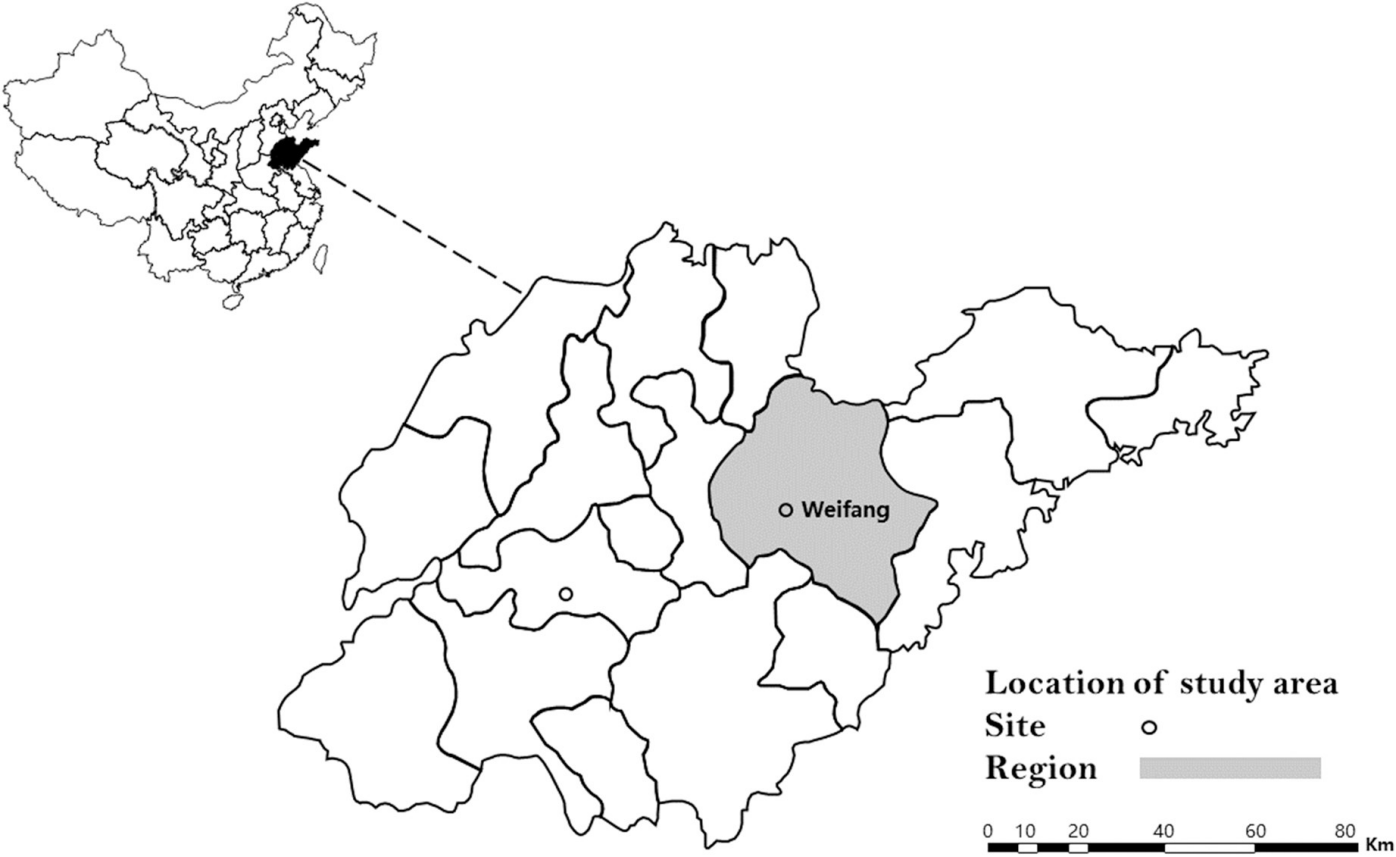
Geographic location of tick collection sites in Shandong Province, China. Location of Shandong Province in China (left) and the location of Weifang city, within the province where tick samples were collected, in 2020. This map was constructed using ArcGIS software, with the base layer sourced from https://www.resdc.cn/data.aspx?DATAID=205. The base layer of the map in this database is shared data and no additional licences are required.

From July through August 2020, tick samples were collected in Weifang city, Shandong Province (118°89’E, 36°68’N) (Fig 1). Sheep were the predominant domestic animals in this area, and the ticks attached to sheep were carefully removed with tweezers. The sheep that were used to collect samples in this study were from the same farm and in addition these sheep were randomly selected from a large flock of sheep. The collected ticks were placed into the collection pipe and stored in a cool and ventilated place, and then transported to the laboratory as soon as possible for further processing. Subsequently, all engorged female ticks were transferred into separate labeled tubes containing moist cotton. They were then kept at a temperature of 28˚C and a humidity level of 80% until they laid eggs and the larvae hatched. The whole egg clutch from each female tick was pooled and the mass of egg clutches in this study ranged from 5 to 35 mg. Each blood-sucking female tick was individually placed in a separate pool. Finally, all tick related samples were stored separately and transferred to the refrigerator for further use.

### Tick identification

**Fig 2:**
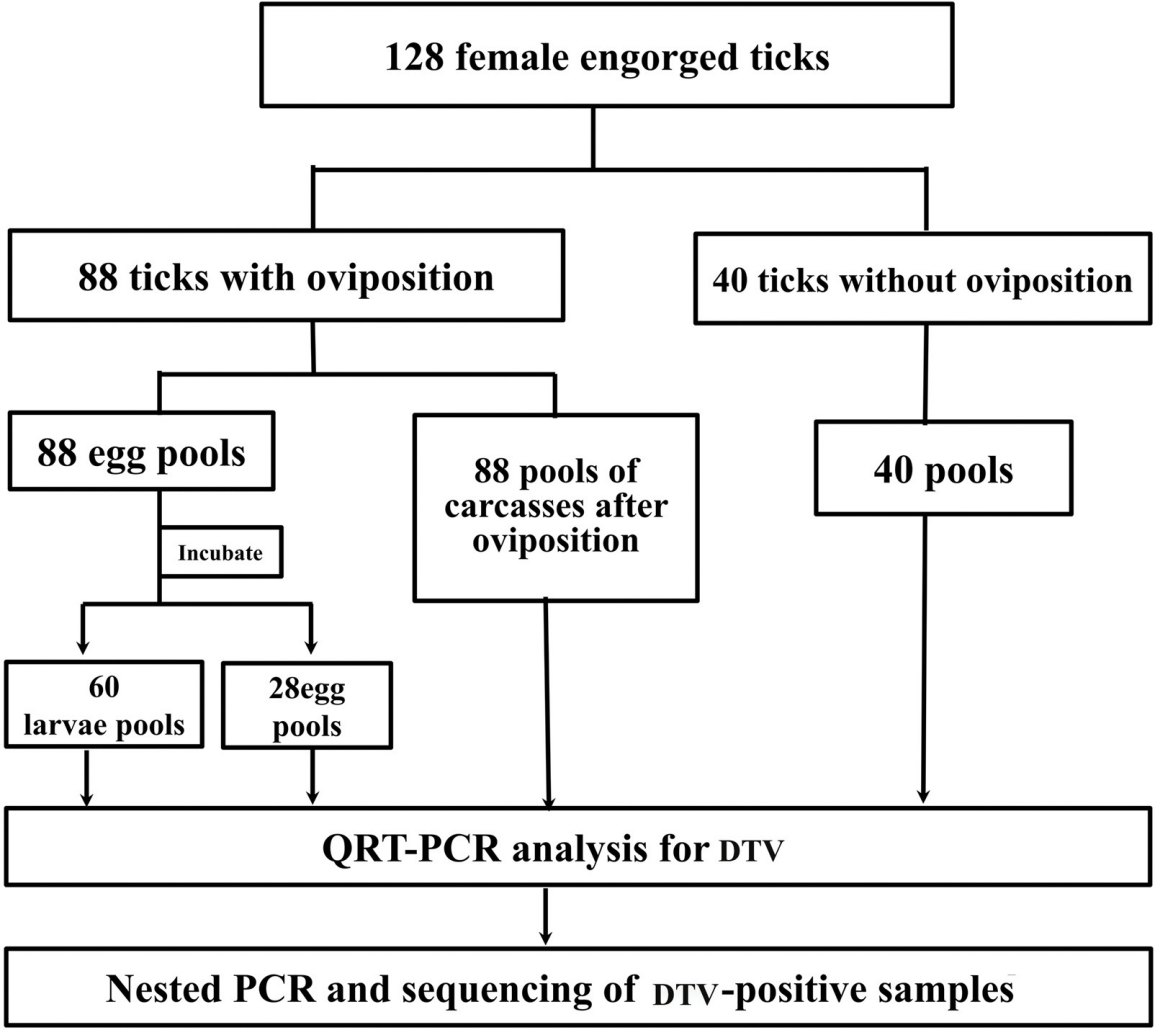
Summary of study design.

This study exclusively included engorged female ticks, which were identified by morphology (Fig 2) and further confirmed by detecting the conserved gene of ticks: *COI*. The PCR primers utilized for *COI* amplification in this study were: LCO1490 (GGT CAA CAA ATC ATA AAG ATA TTG G) and HCO2198 (TAA ACT TCA GGG TGA CCA AAA AAT CA). The reaction was 20 μL, containing 10μL primer STAT, 6μL RNase-free water, 1μL primer, 2μL templates. The PCR reaction conditions were 98°C for 10sec, 55°C for 5sec, 72°C for 2min10sec, and the reaction lasted for 40 cycles.

### RNA extraction and cDNA synthesis

The female ticks, eggs and hatchlings were soaked in 75% alcohol for 30 min and then rinsed with sterile water to remove possible contamination from the body surface of the samples. In the meantime, samples were digested with 10 U RNase I (Thermo Fisher Scientific, USA) at 37˚C for 30 min and then rinsed again with sterile water and air dried on sterile dishes. QRT-PCR was performed on all female engorged ticks and their offspring to determine the presence of DBTV. Each tick specimen was placed in a clean 1.5 mL centrifuge tube and homogenized with 500 μL of frozen Dulbecco’s modified eagle medium (DMEM). The homogenate was centrifuged at 10,000 g and 4˚C for 10 min, and total RNA was extracted from the supernatant using the TIANamp RNA Extraction Kit (Tiangen, China). Complementary DNA (cDNA) of total RNA was synthesized using the Transcriber High Fidelity cDNA Synthesis Kit (Roche, Germany) according to the manufacturer’s instructions. Total RNA and synthesized cDNA were stored at -80˚C until use.

### PCR for detection of DBTV in ticks

QRT-PCR was performed to confirm the presence of DBTV, and nested PCR was applied to amplify positive samples to obtain sequence information. In this study, the primers and probes used to detect DBTV RNA by qRT-PCR have been previously described: DBTV-F (TGC TCC TCT CCG CAC ACC T), DBTV-R (TGG CAA GTA GAG GAA ACT GGT GA) and DBTV-P (FAM-TCC CTC CAG CCA TCA CCA CCT CC-BHQ1) [12]. The reaction was carried out in a volume of 30 μL, containing 6μL 5× one step U^+^ Mix, 1.5μL one step U^+^ Enzyme mix, 0.6μL 50× ROX Reference Dye, 15.4μL RNase-free water, 0.6μL primer and, 0.3μL probe, 5μL RNA. And the qRT-PCR was performed at 55°C for 15 min, 95°C for 30 sec, followed by 40 cycles of 95°C for 10 sec, 60°C for 30 sec. The cycle threshold (Ct) value for a positive sample was set at 35 cycles. In addition, all positive samples by qRT-PCR were further validated by nested PCR using primer sets: Out-F (GGC AGC ACT TTC ACG GAT G), Out-R (CCC CTG TCA TGT CTA ATC AAT GG), In-F (GCA AGC AGA GCC TCA AGA AGC) and In-R (GCC AGA TTG CGA TCC AAG TAT G) [12]. The reaction was carried out in a volume of 20 μL, containing 10μL primer STAT, 6μL RNase-free water, 1μL upstream primer and downstream primer, 2μL templates. The nested PCR reaction conditions were 98°C for 10sec, 55°C for 5sec, 72°C for 2min10sec and 98°C for 10sec, 55°C for 5sec, 72°C for 1min20sec, and the reaction lasted for 40 cycles. The amplified PCR products were purified by 1% agarose gel electrophoresis and visualized by the ChemiDoc imaging system (Bio-Rad, USA). High-quality amplification products were sent to Shanghai Sangon Biotech Company for Sanger sequencing.

### Virus isolation

In this study, Vero, BHK-21 and C6/36 cells were used to isolate DBTV. Briefly, all DBTV-positive tick homogenates were filtered and inoculated on monolayers of Vero, BHK-21 and C6/36 cells. Vero and BHK-21 cells were cultured at 37°C with 5% CO_2_ and C6/36 cells were cultured at 30°C with 3% CO_2_. All samples were blindly passaged for three generations. The cell supernatants from each generation were collected and qRT-PCR was performed to detect the viral content in the supernatants.

### Data analyses

**Fig 3:**
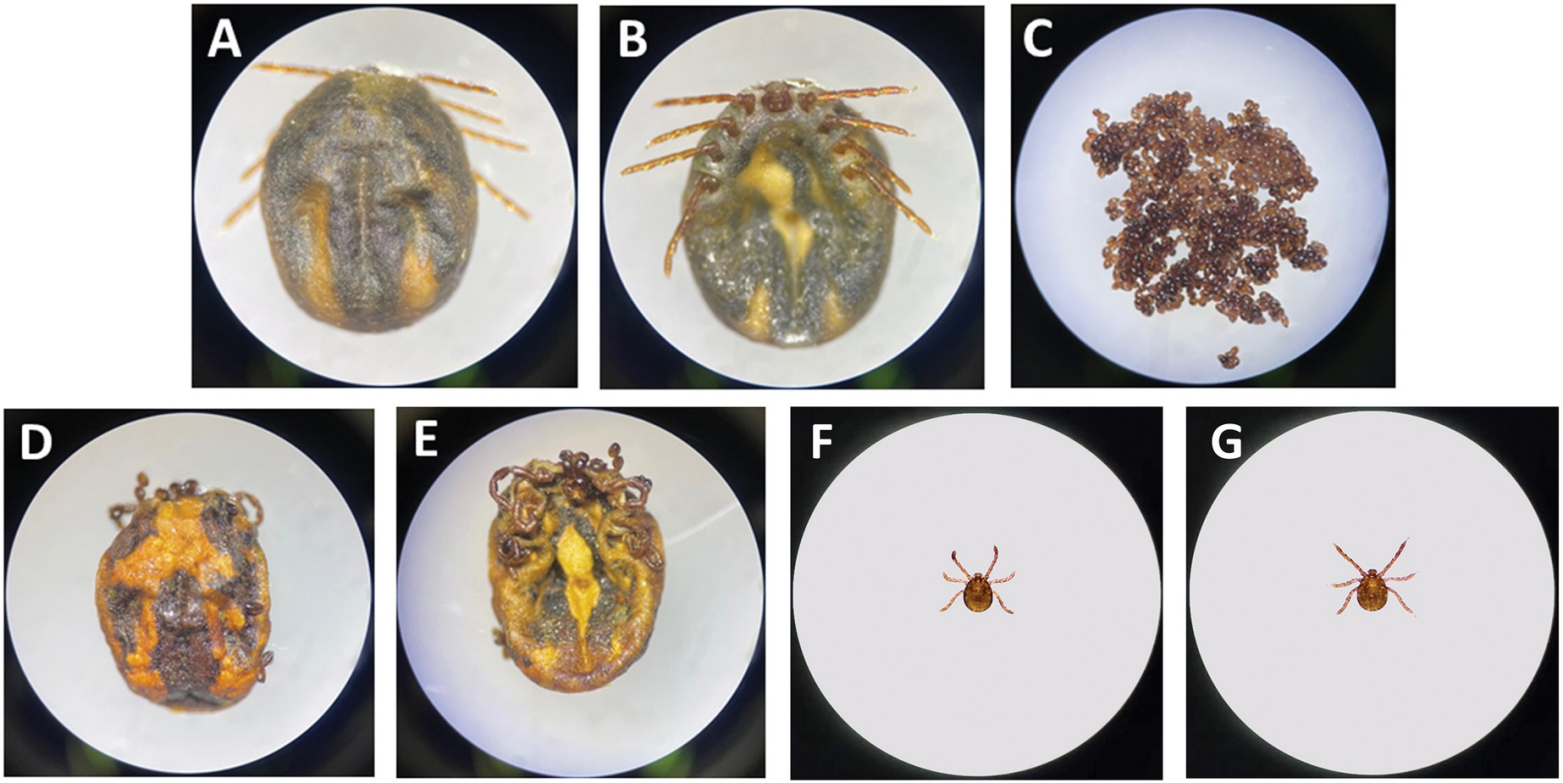
Light microscopic photographs of ticks involved in this study. A: Dorsal view of ticks before oviposition; B: Ventral view of ticks before oviposition; C: Egg masses; D: Dorsal view of ticks after oviposition; E: Ventral view of ticks after oviposition; F: Ventral view of larvae tick; G: Dorsal view of larvae tick.

The prevalence of DBTV in ticks was calculated using the positive pool/total pool method. Differences between positive rates were statistically analyzed using Fisher’s exact test, and differences were considered statistically significant when P<0.05. The DBTV sequences obtained for this study and available in Genbank were compared and analyzed using SnapGene 2.3.2 (GSL Biotech LLC, USA). Phylogenetic trees were constructed using MEGA 5.1 software (MEGA, New Zealand) and the topology of the trees was evaluated using the neighbor-joining method with 1,000 replicates. The operation route of our experimental design is shown in the Fig 3.

## Results

### Tick collection

In this study, 128 blood-sucking female ticks were collected from 15 sheep on the same farm in Weifang city, Shandong Province from July to August 2020. All tick samples were morphologically identified as *H*. *longicornis*, and PCR amplification of the *COI* gene in all tick samples yielded DNA bands of uniform size. Sequencing and analysis of the PCR products showed that all ticks were *H*. *longicornis*. Out of 128 ticks, the 88 ticks laid eggs, while the remaining 40 ticks did not lay eggs. Then, all blood-sucking female ticks and their eggs were sorted into separate test tubes (pool = 216) for nucleic acid extraction and RNA detection.

### Detection of DBTV RNA in ticks

QRT-PCR results showed that RNA of DBTV was detected in engorged female ticks, larvae, and eggs in this study. The positive rate of DBTV was 6.25% (8/128) (95%CI: 1.3%-11.2%) in engorged female ticks, 3.57% (1/28) (95%CI: 0–19.5%) in eggs and 5% (3/60) (95%CI: 0–15.15%) in larvae pools, respectively. The pools of DBTV-positive engorged female ticks were further comprised of 6 pools of post-oviposited ticks (6/88; 6.82%) (95%CI: 0–13.8%) and 2 pools of non-oviposited ticks (2/40; 5%) (95%CI: 0–14.7%). Fisher’s exact test was conducted to evaluate the differences of DBTV prevalence in female engorged ticks and eggs. There was no significant difference in DBTV prevalence between eggs and engorged female ticks with *P*>0.9999. Notably, DBTV was detected in six egg-laying *H*. *longicornis*, while one egg mass and three offspring larvae from these DBTV-positive ticks were also positive for DBTV simultaneously. Unfortunately, DBTV was not successfully isolated from any positive tick samples.

### Phylogenetic analysis

In this study, 5 partial S segments of DBTV were successfully amplified and sequenced from 12 qRT-PCR positive pools, 1 from unoviposited tick, 2 from oviposited ticks and 2 from the corresponding larvae pools. The sequences have been uploaded to the GenBank database with the accession numbers MZ965042- MZ965046.

**Fig 4:**
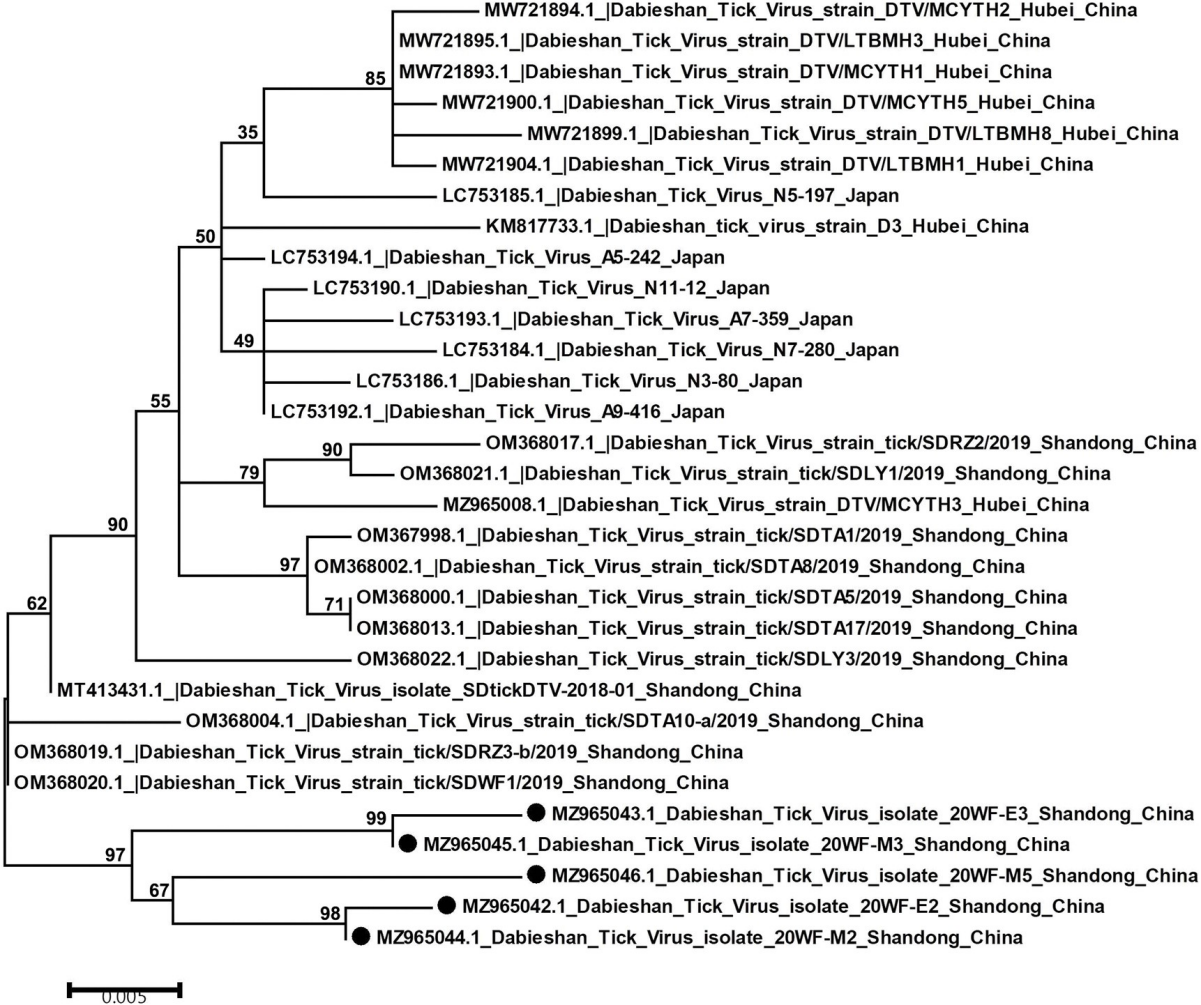
Phylogenetic analysis of DBTV S-segment sequences amplified from ticks and larvae. A phylogenetic tree based on S-segment sequences (548bp) by the maximum likelihood (ML) method using MEGA 5.1 is shown. The numbers above the branches indicate bootstrap values. Black circles indicate DBTV sequences identified in the current study.

Phylogenetic analyses were executed and the result indicated that DBTV was evolutionarily closely related to Yongjia tick virus and Okutama tick virus, as same as previously reported [12]. In addition, a phylogenetic tree was constructed using the maximum likelihood method based on the 548 nt S-segment sequence obtained in this study, as well as DBTV sequences already identified from ticks of Hubei and Shandong provinces in China and some regions in Japan (Fig 4). Based on the results, all available DBTV sequences could be divided into two major phylogenetic groups. The first group contained virus samples from Shandong province and Hubei province of China together with DBTV from Japan, whereas the second group only consisted of DBTV identified in the study. Within the first group, the viruses could be further divided into two subgroups. The DBTV strains from Hubei province of China clustered with viruses from Japan to constitute phylogenetic subgroup I, while some DBTVs isolated from Shandong province and a DBTV strain identified from Hubei province composed the subgroup II. In the second group, the five sequences obtained in this study can be observed clustered together, showing a very close evolutionary relationship with each other. In general, the S segment sequences of DBTVs from different regions showed relatively high sequence identity. A pairwise distance analysis showed that the sequences obtained in this study had 96.7–99.6% nucleotide identity and 95–98.5% identity with sequences reported from other countries and regions, indicating a close evolutionary relationship between these DBTV isolates.

## Discussion

The latest information on the classification of viruses from the International Committee on Taxonomy of Viruses (ICTV) 2022, DBTV is classified in the genus Uukuvirus, Phenuiviridae. It was shown that DBTV clustered together with Yongjia tick virus, Uukuniemi virus and Okutama tick virus in the phylogeny and was widespread in ticks from Shandong Province and Zhoushan Islands of China [12, 18]. However, the distribution of this virus may be broader, as *H*. *longicornis* is one of the most common tick species in China and is distributed throughout the country. To date, the transmission route of this novel virus and its pathogenicity to humans and animals were unknown due to the lack of relevant studies. Therefore, more attention and investigation of DBTV is necessary. There are multiple routes of transmission of TBVs, among which transovarial transmission is a very important one. Although ticks can transmit some specific pathogens via eggs in a simulated laboratory environment, the actual effect of transovarial transmission may be blocked or reduced due to variations or lack of appropriate reservoirs or susceptible hosts under natural conditions [19]. Consequently, studies through observing the transovarial transmission of DBTV in a natural state have a critical role in contributing to the ecology of this virus.

Compared with the DBTV prevalence in unfed ticks in Weifang (0.67%) described previously, we found a high prevalence of this virus in engorged female ticks (6.25%) from domestic sheep in this area [12]. At the same time, it is important to note that the sheep came from areas adjacent to the sampling sites of the previous study, and the 15 sheep sampled were randomly selected from the flock. This high prevalence of the virus might imply that blood feeding can promote virus proliferation or play an active role in virus transmission. Furthermore, unlike the woolen flannel cloth dragging method, ticks were collected directly from domestic sheep in this study. Thus, differences in sampling methods may also result in differences in viral positive rates. Importantly, DBTV was detected simultaneously in most of the engorged female ticks and their offspring, suggesting that DBTV could be transmitted transovarially in *H*. *longicornis*. Furthermore, our results verified the possibility of *H*. *longicornis* as a competent vector in the natural circulation of DBTV and also displayed the transmission mode of the virus in tick populations. However, DBTV was not detected in a portion of the pool of offspring of DBTV-positive engorged ticks, perhaps because the low viral load transmitted from maternal ticks to egg resulted in too few copies of DBTV to reach the detection threshold. In addition, too few egg mass pools were obtained from DBTV-positive oviposited females in this study; therefore, it is imperative to collect more data concerning ticks laying eggs under natural environment and artificial simulation conditions to further confirm the transovarial transmission of DBTV in ticks.

Phylogenetic analysis was conducted by constructing a phylogenetic tree based on the nucleotide sequences of the S segment obtained in this study and other reference sequences from GenBank. The result indicated that the 5 DBTV strains in this study clustered with the sequences found in Shandong Province, Hubei Province and Japan, suggesting that the DBTV sequences among these remote regions did not change much. These results show that DBTV species are highly conservative and its evolution in nature is relatively slow, which was also consistent with our previous research results. However, it should be further verified by isolating the virus and obtaining the whole genome sequence. Interestingly, the isolate called 20WF-E2 from the larvae pool shared almost the same identity (99.6%) with 20WF-M2 from its maternal tick. At the same time, similar results appeared in another group, that is, the isolate 20WF-E3 from larvae grouped more closely with 20WF-M3 from maternal tick. These signified a potential link of DBTV between engorged female ticks and their eggs, and DBTV may be transmitted from infected female ticks to its progeny.

In addition, the limitations of this study should not be ignored. Firstly, owing to the centralized processing of tick eggs, it is difficult to ascertain the exact proportion of transovarial transmission from DBTV-infected maternal ticks to their eggs. Secondly, we are unable to discuss DBTV infection in subsequent life stages of ticks only by inspecting the infection status of egg masses and larvae. Thirdly, this study failed to isolate the live virus of DBTV. This may be due to the titer of the live virus in the sample being too low or because a suitable cell line is not available.

In brief, our research provided molecular epidemiological evidence for the prevalence of DBTV in ticks of an endemic region and suggested that DBTV may be transmitted vertically from infected adult female ticks to eggs through transovarial transmission. *H*. *longicornis* may be an important vector of DBTV and play a crucial role in the transmission cycle of the virus.
